# Prophylactic exercises among head and neck cancer patients during and after swallowing sparing intensity modulated radiation: adherence and exercise performance levels of a 12-week guided home-based program

**DOI:** 10.1007/s00405-016-4367-9

**Published:** 2016-11-03

**Authors:** Ingrid C. Cnossen, Cornelia F. van Uden-Kraan, Birgit I. Witte, Yke J. Aalders, Cees J. T. de Goede, Remco de Bree, Patricia Doornaert, Derek H. F. Rietveld, Jan Buter, Johannes A. Langendijk, C. René Leemans, Irma M. Verdonck-de Leeuw

**Affiliations:** 1 Department of Otolaryngology, Head and Neck Surgery, VU University Medical Center, PO Box 7057, 1007 MB Amsterdam, The Netherlands; 2 Department of Clinical Psychology, VU University, Amsterdam, The Netherlands; 3 Department of Epidemiology and Biostatistics, VU University Medical Center, Amsterdam, The Netherlands; 4 Department of Rehabilitation Medicine and Physical Therapy, VU University Medical Center, Amsterdam, The Netherlands; 5 Department of Head and Neck Surgical Oncology, UMC Utrecht Cancer Center, University Medical Center Utrecht, Utrecht, The Netherlands; 6 Department of Radiation Oncology, VU University Medical Center, Amsterdam, The Netherlands; 7 Department of Medical Oncology, VU University Medical Center, Amsterdam, The Netherlands; 8 Department of Radiation Oncology, University Medical Center Groningen, University of Groningen, Groningen, The Netherlands

**Keywords:** Head and neck cancer, (Chemo)radiation, Prophylactic exercises, Swallowing problems, Speech problems

## Abstract

The background and purpose of this paper is to investigate adherence, exercise performance levels and associated factors in head and neck cancer (HNC) patients participating in a guided home-based prophylactic exercise program during and after treatment [swallowing sparing intensity modulated radiation therapy (SW-IMRT)]. Fifty patients were included in the study. Adherence was defined as the percentage of patients who kept up exercising; exercise performance level was categorized as low: ≤1, moderate: 1–2, and high: ≥2 time(s) per day, on average. Associations between 6- and 12-week exercise performance levels and age, gender, tumour site and stage, treatment, intervention format (online or booklet), number of coaching sessions, and baseline HNC symptoms (EORTC-QLQ-H&N35) were investigated. Adherence rate at 6 weeks was 70% and decreased to 38% at 12 weeks. In addition, exercise performance levels decreased over time (during 6 weeks: 34% moderate and 26% high; during 12 weeks: 28% moderate and 18% high). The addition of chemotherapy to SW-IMRT [(C)SW-IMRT] significantly deteriorated exercise performance level. Adherence to a guided home-based prophylactic exercise program was high during (C)SW-IMRT, but dropped afterwards. Exercise performance level was negatively affected by chemotherapy in combination with SW-IMRT.

## Introduction

Intensity modulated radiation therapy (IMRT) targeting head and neck cancer (HNC) patients allows for more conformal dose distribution, aiming to minimize the dose to the surrounding healthy tissues and to spare normal structures (i.e. the parotid glands). Treatment with IMRT has proven to lead to less treatment-related side-effects, such as xerostomia, and to improved health-related quality of life (HRQOL) [1–12]. Attempts are made to also spare other organs at risk (OARs), such as the submandibular glands [13], and the swallowing structures [14]. Van der Laan et al. [1, 14] demonstrated that, compared with the standard IMRT, reduction of the dose to the swallowing OARs (SWOARs) has the potential to reduce the risk on swallowing problems through swallowing sparing IMRT (SW-IMRT). It is hypothesized that patients should be encouraged to maintain oral intake and to perform exercises to promote the use of the muscles in the head and neck area. The ongoing use of the swallowing, speech, and shoulder mechanisms during and after treatment may enhance the potential benefits of SW-IMRT [15, 16]. Therefore, we developed a guided home-based prophylactic exercise program ‘Head Matters’ to maintain muscle structure and swallowing, speech, and shoulder function (HM) [17]. Offering HNC patients such a prophylactic exercise program may delay the decline of lean muscle mass in the head and neck area, and may limit the extent of post-treatment impairment [15, 18–29], eventually leading to improved HRQOL [15, 16, 21–27, 30]. The current literature on prophylactic exercise programs varies considerably in terms of timing, intensity, duration, frequency, and type of exercise. In addition, a broad range (13–71%) of adherence rates has been reported [17, 21, 25, 31–33]. However, information on patient’s adherence to home-based exercise programs, on data collected related to daily exercise performance, and on factors that could potentially influence patient’s exercise performance is lacking. How realistic an approach is regarding home-based exercise programs in HNC patients during SW-IMRT is unknown. Therefore, the purpose of this study was (1) to investigate adherence to a 12-week home-based exercise program during SW-IMRT, (2) to investigate exercise performance levels, (3) to investigate whether demographic and clinical factors, or HNC-specific HRQOL at baseline is associated with exercise performance levels, and (4) to investigate whether exercise performance levels are associated with the course of HNC-specific HRQOL during the entire 12-week exercise program.

## Materials and methods

### Design

A prospective clinical cohort study.

### Patients

Between 2011 and 2013, HNC patients were included in this study if they were planned for SW-IMRT at VU University Medical Center (VUmc), Amsterdam, The Netherlands. Patients fulfilled the following criteria: (1) age ≥ 18 years, (2) cancer originating in the oral cavity, oropharynx, hypopharynx, or larynx, (3) SW-IMRT alone or in combination with chemotherapy [(C)SW-IMRT], (4) performance status 0–2 on the World Health Organization Scale [34], (5) the absence of severe cognitive impairment, and (6) sufficient mastery of the Dutch language (criteria 4–6 as judged by the radiation oncologist who included the patients in this study). Patients who previously underwent surgery, radiotherapy, or chemoradiation, who had prior malignancies in the head and neck area, and/or distant metastases were excluded. Patients with physician-rated RTOG grade 2–4 swallowing dysfunction at baseline (1 = mild fibrosis, slight difficulty in swallowing solids, no pain in swallowing; 2 = unable to take solid food normally, swallowing semi-solid food; 3 = severe fibrosis, able to swallow only liquids, may have pain in swallowing; 4 = necrosis, complete obstruction) (according to the RTOG/EORTC Late Radiation Morbidity Scoring Schema [35]) were also excluded to ensure that the observed swallowing dysfunction was induced by radiation treatment itself and not by tumour extension.

Patients were treated with curative intent using (C)SW-IMRT. In all patients, parotid glands and swallowing structures were spared when possible, without compromising the dose to the target volumes. A simultaneous integrated boost technique was used with bilateral elective irradiation of the neck nodes to a total dose of 57.75 Gy, using a dose per fraction of 1.65 Gy. The primary tumour and pathological lymph nodes were treated to a total dose of 70 Gy, in fractions of 2 Gy. Chemotherapy was given concurrently with radiotherapy and consisted generally of cisplatin 100 mg/m^2^ intravenously on days 1, 22, and 43.

The study was approved by the ethical committee of the VU University Medical Center Amsterdam. Written informed consent was obtained from all participating patients.

### Intervention

The guided home-based exercise program Head Matters (HM) was developed by speech and swallowing therapists, physiotherapists, head and neck surgeons, and radiation oncologists. HM was based on the previous research [15, 16, 19–30] and on clinical practice. HNC patients were recommended to perform HM exercises for at least 15 min per day in total. HM is comprised of the following prophylactic exercises: (1) exercises to maintain mobility of the head, neck, and shoulders (e.g., ‘Moving shoulders up and down’, ‘Circling shoulders forward and backward’) (‘Shoulder’), (2) exercises to optimize and maintain swallowing function (e.g., ‘Swallowing with strength: effortful swallow’, ‘Taking sips of water regularly’ (‘Swallow’), (3) exercises to optimize and maintain vocal health and vocal function (e.g., ‘Humming with gradually increased volume, and with exaggerated jaw movement’, ‘Slide up the pitch scale as high as possible’ (Falsetto exercise) (‘Voice’), and (4) exercises to optimize and maintain speech function and functional communication (e.g., ‘Articulate each syllable’, ‘Stretching the tongue out straight’(‘Speech’). HM informs the patient on possible swallowing, speech, and shoulder problems during treatment, and encourages patients to perform exercises to maintain function [17]. Based on our clinical experience and earlier study [17], we encourage patients to exercise at least once a day for 15 min and preferably three times a day. HM is available in two different formats: (a) online [36] with a description of the exercises, and with photo and video examples of the exercises, (b) a 28-page booklet, with the same information as the online version, photo examples of the exercises, and a 15-min instructional DVD with video examples of the exercises. Patients can choose the format that fits their needs best.

Before patients carry out HM at home, a 15-min face-to-face instruction session with expert speech and swallowing therapist’s demonstration of the exercises is planned on the first day of (C)SW-IMRT. During the course, each patient is contacted by phone in a weekly 10-min coaching session by an experienced speech therapist. Patients are asked to fill out a diary on paper or online for 12 weeks. In their diaries, patients note which exercises (of the four exercise categories) they performed, and the frequency of exercising (1, 2, or 3 times per day).

### Measures

A study specific survey was composed comprising items on sociodemographic data (age, gender, HM format, and number of coaching sessions) and on HNC-specific HRQOL (EORTC-QLQ-H&N35) [37]. This survey was assessed at baseline (T0), every week from the 1st till the 6th week of treatment with (C)SW-IMRT (T1-T6), and 6 weeks after the end of treatment (T12). Clinical data (tumour site, tumour stage, and treatment modality) were abstracted from the hospital information system.

#### Adherence and exercise performance levels

Adherence concerned the percentage of patients who started and kept up with the HM exercise program at least once a day across the 6-week period during treatment with (C)SW-IMRT and across the 12-week period during and after treatment with (C)SW-IMRT. Adherence was assessed using patient-completed diaries. Non-adherence was defined as failure to perform any of the exercises. To gain insight into which exercises were performed most often, patient’s diaries were analyzed in more detail regarding the frequency of exercising, exercise performance levels per week during 6 weeks while undergoing treatment, and during 12 weeks during and after treatment. Exercise performance was based on patient diaries and consisted of low-, moderate-, and high-performance levels during 6 and 12 weeks, respectively: (1) low, indicating an exercise performance of all exercise categories at most once a day on average (range 1–168; range 1–336), (2) moderate, indicating an exercise performance of all categories between once and twice a day on average (range 169–336; range 337–672), and (3) high, indicating an exercise performance of all exercise categories at least twice a day on average (range 337–504; range 673–1008). To gain insight into which exercise category was performed most often, the diaries were analyzed in detail regarding the exercise frequency per day on average (1–3 times), the exercise frequency per week (the total number of exercise performed per week ranged from 0 to 84 (4 exercise categories 3 times per day for 7 days), and type of exercise (‘Shoulder’, ‘Swallow’, ‘Voice’, and ‘Speech’).

#### Factors associated with exercise performance level

Data were collected on gender, age, tumour site (oral cavity, oropharynx, hypopharynx, larynx), tumour stage (I, II, III, IV), treatment modality (SW-IMRT or CSW-IMRT), intervention format (online or booklet), coaching (number of sessions), and on HNC-specific HRQOL (EORTC-QLQ-H&N35).

### Statistical analysis

Descriptive statistics were used to summarize adherence, exercise performance levels, number of coaching sessions, demographic and clinical characteristics, and HNC-specific HRQOL (EORTC-QLQ-H&N35). A Chi-square test was used to examine differences in exercise performance level at 6 and 12 weeks (low vs moderate/high), regarding gender (male vs female), tumour site (oral cavity/oropharynx vs hypopharynx/larynx), tumour stage (stage I/II vs stage III/IV), treatment modality (RT vs CRT), and intervention format (online vs booklet). Fisher’s exact tests were used when the assumption of the expected value of each cell of 5 or higher was not met. Independent samples *t* tests were used to investigate differences in exercise performance level at 6 and 12 weeks (low vs moderate/high) regarding age, and Mann–Whitney *U* tests regarding the number of coaching sessions (at 6 or 12 weeks), and HNC-specific HRQOL at baseline (EORTC-QLQ-H&N35). Longitudinal analysis was performed by generalized estimating equations (GEEs) (jointly testing the bivariate effect of variables and its time dependence) with a logit link function and autoregressive correlation matrix of the first order [AR(1)]. Longitudinal changes in exercise performance level (low vs moderate/high) per week in relation to each of the symptom subscales of the EORTC-QLQ-H&N35 were analyzed. HNC-specific HRQOL was measured weekly from baseline through week 6, and at the end of week 12. The model included both the current value of the symptom subscales as well as the lagged value (i.e. the value of the symptom subscale at the previous assessment) of the symptom subscale. Confounding factors (e.g., number of coaching sessions) were added as fixed effects in the model. Data were analyzed using IBM SPSS Statistics for Windows, version 22. For all analyses, *p* < 0.05 was considered statistically significant.

## Results

### Participants

Ninety-seven patients were eligible during the study period. Thirty-seven patients did not participate (38%). Of these 37 patients, 19 were not willing to participate, 12 refused to fill out any questionnaires, and 6 declared to be too tired. Of 60 patients who performed the exercises, 10 diaries were not available, leaving a study sample of 50 patients. Table 1 shows the demographic, tumour, and treatment characteristics of the study population.

**Table 1:** Demographic and clinical characteristics (*n* = 50)

Age	
Mean age, years (range)	61 (40–77)
	*N* (%)
Gender	
Male	39 (78)
Female	11 (22)
Tumour site	
Oropharynx	30 (60)
Larynx	15 (30)
Hypopharynx	5 (10)
Tumour stage	
I	4 (8)
II	3 (6)
III	17 (34)
IV	26 (52)
Treatment	
SW-IMRT	23 (46)
CSW-IMRT	27 (54)
HM format	
Online	26 (52)
Booklet	24 (48)
12-week coaching sessions	
Median (range)	9 (4–12)

### Adherence

Table 2 shows that of 50 patients, 35 patients started and kept up exercising across the first 6 weeks (6-week adherence rate of 70% and 19 patients kept up exercising up to 12 weeks (12-week adherence rate of 38%).

**Table 2:** Participant’s weekly and 12-week exercise performance levels (*n* = 50)

Patient number	Format	Week number												Total number of exercises performed	12-week exercise performance level
		1	2	3	4	5	6	7	8	9	10	11	12		Low (1–336)
73	ONLINE	0	0	0	4	0	0	0	0	0	0	0	0	4	
9	ONLINE	0	0	8	0	0	0	0	0	0	0	0	0	8	
74	ONLINE	0	0	0	0	12	0	0	0	0	0	0	0	12	
106	BOOK	0	12	0	0	0	0	0	0	0	0	0	0	12	
76	BOOK	15	0	0	0	0	0	0	0	0	0	0	0	15	
132	ONLINE	0	0	8	4	4	0	0	0	0	0	0	0	16	
44	BOOK	0	0	11	11	9	5	7	6	5	6	7	6	73	
69	BOOK	12	23	24	16	0	0	0	0	0	0	0	0	75	
154	ONLINE	16	14	25	21	6	7	0	0	0	0	0	0	89	
150	ONLINE	0	8	4	12	8	0	16	16	12	16	4	0	96	
40	ONLINE	0	0	0	29	21	3	15	17	15	12	0	0	112	
183	BOOK	34	7	10	8	7	7	7	7	7	7	7	7	115	
155	BOOK	12	12	6	11	12	12	13	11	12	11	11	9	132	
109	ONLINE	44	12	0	0	0	0	0	0	0	0	40	56	152	
96	BOOK	3	8	9	1	32	31	4	17	20	15	18	19	177	
151	BOOK	8	28	16	17	14	14	14	15	18	18	18	14	194	
80	BOOK	28	36	40	12	4	8	4	16	24	12	20	16	220	
38	ONLINE	24	40	48	44	48	40	0	0	0	0	24	0	268	
149	BOOK	40	47	28	48	36	28	0	0	28	0	12	4	271	
137	BOOK	16	28	29	25	26	30	17	34	30	28	23	0	286	
95	BOOK	18	24	26	18	28	19	24	24	28	24	25	28	286	
78	BOOK	29	39	36	32	28	32	25	1	4	10	28	28	292	
55	ONLINE	12	63	48	51	20	20	12	8	4	16	28	28	310	
130	ONLINE	34	34	49	41	34	15	30	49	15	0	12	0	313	
45	BOOK	48	56	56	56	56	24	20	0	0	0	0	0	316	
37	BOOK	36	84	84	36	24	56	0	0	0	0	0	0	320	
110	ONLINE	24	28	28	16	28	28	28	28	28	28	28	28	320	
107	ONLINE	32	12	32	50	28	25	33	43	25	27	23	20	350	Moderate (337–672)
71	ONLINE	21	72	61	64	47	50	44	27	0	0	0	0	386	
97	ONLINE	48	56	52	40	36	36	32	8	0	36	32	28	404	
75	BOOK	36	84	56	56	56	56	0	0	12	28	28	28	440	
57	ONLINE	28	56	52	58	52	40	28	28	36	20	28	24	450	
170	BOOK	72	84	84	72	72	69	0	0	0	0	0	0	453	
134	BOOK	57	63	67	60	60	66	47	44	0	0	0	0	464	
39	BOOK	0	0	48	48	48	48	40	56	48	48	48	48	480	
94	ONLINE	44	68	54	46	28	32	36	30	36	56	56	56	542	
118	ONLINE	64	84	84	84	84	69	63	16	0	0	0	0	548	
22	BOOK	48	70	28	28	28	12	0	0	84	84	84	84	550	
14	ONLINE	39	55	66	56	59	46	17	25	42	50	53	49	557	
186	ONLINE	63	66	60	48	60	54	55	53	46	46	9	0	560	
187	BOOK	72	84	84	84	84	76	67	52	8	0	0	0	611	
93	ONLINE	0	64	80	84	80	68	53	28	36	36	64	80	673	High (673–1008)
168	ONLINE	0	28	36	84	84	81	63	63	63	63	63	63	691	
129	ONLINE	0	24	50	76	80	84	43	38	84	80	84	84	727	
20	BOOK	48	84	42	42	84	72	84	84	84	84	84	84	876	
182	ONLINE	52	80	84	84	66	60	64	75	79	80	76	84	884	
133	ONLINE	64	84	80	80	72	84	60	84	84	84	64	44	884	
72	BOOK	72	84	84	84	84	75	75	84	84	84	84	84	978	
167	BOOK	60	84	84	84	84	84	84	84	84	84	84	84	984	
70	ONLINE	72	84	84	84	84	84	84	84	80	84	84	84	992	

0 = non-active (does not perform any exercises)

84 = high performance (i.e., four exercise categories three times per day for 7 days)

### Exercise performance level

Table 2 presents the 12-week exercise performance levels, and exercise performance levels per week of all 50 individual patients.

Of all 50 patients, 20 patients (40%) had a low 6-week exercise performance level, 17 (34%) had a moderate, and 13 (26%) had a high exercise performance level.

Of all 50 patients, 27 patients (54%) had a low 12-week exercise performance level, 14 (28%) had a moderate, and 9 (18%) had a high exercise performance level).

**Fig. 1:**
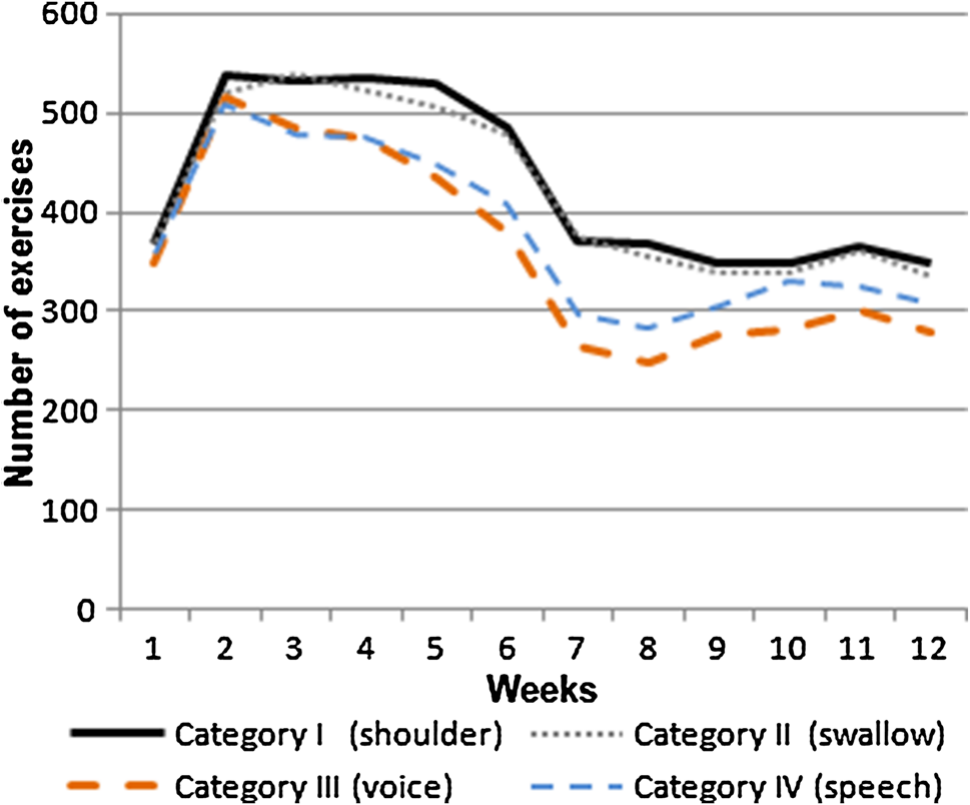
Total number of weekly performed exercises by category (*n* = 50)

Figure 1 presents the weekly exercise performance by exercise category. At the 6th and the 12th week, respectively, patients most often (484 and 348 times) performed the exercises to maintain mobility of the head, neck, and shoulders, and the exercises and strategies to optimize, and to maintain swallowing function: 477 and 336 times.

### Factors related to exercise performance levels

Table 3 shows the 6- and 12-week exercise performance levels in relation to demographic (age, gender) and clinical factors (tumour site, tumour stage, and treatment modality), HM intervention format, and to the median number of coaching sessions. Significantly, more patients treated with chemotherapy (CSW-IMRT) had a low exercise performance level over the first 6 weeks compared with patients who were treated with SW-IMRT alone, *χ* ^2^(1, *N* = 50) = 5.92, *p* = 0.15 as well as over the entire 12 weeks, *χ* ^2^(1, *N* = 50) = 13.36, *p* < 0.001. Exercise performance levels during 6 and 12 weeks were not significantly associated with age, gender, tumour site, tumour stage, HM intervention format, or number of coaching sessions. HNC-specific HRQOL at baseline was not associated with exercise performance level during or after treatment (Table 4). Changes in exercise performance levels per week in relation to the value of the EORTC-QLQ-H&N35 subscales in the previous week were analyzed, using generalized estimating equations (GEEs). Exercise performance level was significantly related to the symptom item ‘Problems with mouth opening’: experiencing more problems with mouth opening in the previous week yielded lower odds for a moderate-to-high exercise performance level in the next week [OR (95% CI) = 0.91 (0.84–0.99), *p* = 0.037 (Table 5)]. This means that the more problems a patient experiences with opening his mouth in the previous week, the more likely it is he will have a lower exercise performance level the next week. However, after correcting for treatment modality (SW-IMRT vs CSW-IMRT), this significant effect of problems with mouth opening disappeared (*p* = 0.16).

**Table 3:** Exercise performance levels in relation to demographic and clinical factors

	Low level after 6 weeks	Moderate-to-high level after 6 weeks	*p* value	Low level after 12 weeks	Moderate-to-high level after 12 weeks	*p* value
	*N* (%)	*N* (%)		*N* (%)	*N* (%)	
	20 (40)	30 (60)		27 (54)	23 (46)	
Age			0.48			0.12
Mean age, years (range)	60 (46–76)	62 (40–77)		59 (40–76)	63 (50–77)	
Gender			0.74			0.97
Male	15 (38)	24 (62)		21 (54)	18 (46)	
Female	5 (45)	6 (55)		6 (54)	5 (46)	
Tumour site			0.56			0.64
Oropharynx	13 (43)	17 (57)		17 (57)	13 (43)	
Larynx/Hypopharynx	7 (35)	13 (65)		10 (50)	10 (50)	
Tumour stage			1.00			0.69
I/II	3 (43)	4 (57)		3 (43)	4 (57)	
III/IV	17 (40)	26 (60)		24 (56)	19 (44)	
Treatment			**0.015**			**<0.001**
SW-IMRT	5 (22)	18 (78)		6 (26)	17 (74)	
CSW-IMRT	15 (56)	12 (44)		21 (78)	6 (22)	
HM format			0.42			0.25
Online	9 (35)	17 (65)		12 (46)	14 (54)	
Booklet	11 (46)	13 (54)		15 (63)	9 (37)	
Coaching sessions			0.18			0.63
Median (range)	5 (3–6)	4 (2–6)		9 (4–12)	9 (4–12)

**Table 4:** Exercise performance levels in relation to HNC-specific HRQOL at baseline

EORTC-QLQ-H&N35	Low level after 6 weeks	Moderate-to-high level after 6 weeks	*p* value	Low level after 12 weeks	Moderate-to-high level after 12 weeks	*p* value
	*N* = 20 (40%)	*N* = 30 (60%)		*N* = 27 (54%)	*N* = 23 (46%)	
	Mean (SD)			Mean (SD)		
Oral pain	26.2 (22.0)	30.3 (28.5)	0.83	27.5 (26.2)	30.1 (26.1)	0.61
Swallowing problems	17.5 (22.1)	20.8 (24.2)	0.52	19.4 (24.8)	19.6 (21.8)	0.70
Sense problems	7.5 (16.6)	3.9 (12.9)	0.18	7.4 (16.8)	2.9 (10.8)	0.20
Speech problems	16.7 (23.8)	22.6 (26.8)	0.31	16.9 (22.7)	24.1 (28.5)	0.27
Social eating problems	10.0 (12.8)	14.2 (21.6)	0.83	13.9 (21.2)	10.9 (15.2)	0.75
Social contact problems	7.3 (10.8)	9.8 (17.6)	0.95	8.4 (14.1)	9.3 (16.5)	0.81
Teeth problems	11.7 (22.4)	22.2 (35.4)	0.38	16.0 (28.3)	20.3 (34.4)	0.81
Mouth opening problems	5.0 (12.2)	14.4 (31.2)	0.51	9.9 (24.1)	11.6 (27.7)	0.99
Dry mouth	10.0 (15.7)	11.1 (22.0)	0.86	11.1 (22.6)	10.1 (15.7)	0.79
Sticky saliva	20.0 (25.1)	12.2 (23.9)	0.18	21.0 (29.4)	8.7 (15.0)	0.16
Coughing	20.0 (19.9)	18.9 (20.9)	0.80	19.7 (19.1)	18.8 (22.1)	0.75
Feeling ill	11.7 (16.3)	16.7 (24.4)	0.61	13.6 (19.1)	15.9 (24.3)	0.86

**Table 5:** Course of HNC-specific HRQOL in relation to weekly exercise performance level

EORTC-QLQ-H&N35	Current value			Lagged value		
	OR	95% CI	*p* value	OR	95% CI	*p* value
Oral pain	1.03	0.94–1.12	0.57	0.93	0.81–1.06	0.26
Swallowing problems	1.07	0.97–1.19	0.19	0.90	0.80–1.01	0.063
Sense problems	1.04	0.92–1.18	0.56	0.94	0.83–1.06	0.31
Speech problems	0.95	0.85–1.07	0.41	0.94	0.84–1.04	0.22
Social eating problems	1.09	0.95–1.24	0.22	0.85	0.71–1.01	0.058
Social contact problems	0.81	0.65–1.02	0.068	1.04	0.89–1.21	0.65
Teeth problems	1.04	0.92–1.17	0.55	0.95	0.86–1.06	0.39
Mouth opening problems	0.95	0.82–1.09	0.43	0.91	0.84–0.99	0.037*
After correcting for treatment	0.96	0.81–1.13	0.59	0.93	0.84–1.03	0.16
Dry mouth	0.97	0.85–1.11	0.70	0.93	0.83–1.03	0.16
Sticky saliva	0.96	0.87–1.07	0.46	0.92	0.81–1.04	0.16
Coughing	1.04	0.95–1.13	0.43	0.91	0.81–1.0	0.080
Feeling ill	0.97	0.87–1.07	0.54	1.00	0.91–1.11	0.99

*OR* odds ratio for moderate/high-performance level per increase of ten points on the subscale

* *p* < 0.05

## Discussion

The key findings of this study are that in HNC patients treated with SW-IMRT alone or in combination with chemotherapy [(C)SW-IMRT] adherence to a guided home-based prophylactic exercise program was high in the first 6 weeks (70%), but dropped after completion of treatment. Exercise performance levels during and after treatment were low especially in patients who were treated with SW-IMRT in combination with chemotherapy.

Few studies have investigated exercise adherence rates among HNC patients during treatment. These studies have yielded inconsistent findings with adherence rates ranging from 13 to 71% [17, 21, 25, 31–33]. This variety of adherence percentages may be a matter of definition. In this study, we used a rather rigid definition of adherence. Adherence was viewed as a dichotomous outcome with a pre-specified threshold value. This means for instance that a patient who was adherent to the program for 6 weeks and took a break from exercise for a week but continued to exercise for the next 5 weeks was defined as non-adherent. Adherence can also be viewed as a categorical or as a continuous outcome (the total number of exercise performed or the percentage of exercises completed [38]). According to Huang [39], only percentage of actual exercise activity over an expected exercise activity, or the number of exercise sessions completed at the prescribed level divided by the total number of exercise sessions prescribed, reflects the essence of adherence. However, the specific timing and the necessary amount of prescribed prophylactic exercises to obtain any therapeutic benefit are largely unknown. In the literature, a gap exists for well-developed measures that capture self-reported adherence to prescribed but unsupervised home-based exercises [40].

Besides insight into adherence to an intervention, it is also interesting to have a closer look on how well patients perform. Our study showed that 40% had a low 6-week exercise performance, while more than half of participants had a low 12-week performance. In a study of Mortensen [32] evaluating the impact of home-based prophylactic swallowing exercises on swallowing-related outcomes in HNC patients treated with curative RT, more patients (53%) than in our study had low (5-week) exercise performance levels. In a retrospective study of Hutcheson [15], 45% of the adherent patients performed the prescribed prophylactic exercises more than four times per day. However, the results of these studies are difficult to compare because of the various categorisations of exercise performance level as outcome measure.

In our study, lower 6- and 12-week exercise performance levels were significantly associated with treatment modality (CSW-IMRT vs SW-IMRT). In addition, we found a progressively downward trend in prophylactic exercise performance, indicating that exercise performance levels were reduced as CSW-IMRT treatment advanced. The previous studies showed an increased symptom burden if chemotherapy was added as treatment modality [5]. HNC patients undergoing CRT experience several toxicities which may result in a reduction of the number of prophylactic exercises completed [41].

A limitation of this study was that the study sample probably consisted of motivated HNC patients who were committed to exercise and who were motivated to complete their exercise diaries also. However, we did not apply a motivational questionnaire, so firm conclusions on the impact of motivation to start exercising cannot yet be drawn. Study results may not be generalizable to a wider population of HNC patients who may feel less motivated. In addition, in this study, we chose to focus on (deterioration of) HNC-specific quality of life outcomes as possible barriers for exercise performance. To evaluate (other) factors possibly associated with exercise performance levels, larger studies should be conducted using objective functional outcome measures in addition to patient-reported outcomes [5, 7], and psychosocial factors [17]. Furthermore, daily exercise behaviour was self-reported by participants and, therefore, may be subject to bias. In an attempt to minimize bias, exercise logs were completed daily. It is not certain, however, that these instructions were followed. The strengths of this study lie in the use of 6- and 12-week adherence data, and data on levels of exercise performance. There is growing evidence of the potential benefits of prophylactic exercises among HNC patients undergoing (C)RT [21, 23–25, 31], but the factors associated with adherence to home-based exercises are largely unknown. Further research is needed to study predictors to improve adherence, such as the perception of illness, the perception of ability to complete therapy, patients’ motivation and intention, behaviours related to home-based exercises, and social support and guidance [42].

## Conclusion

Adherence of HNC patients to a guided home-based prophylactic exercise program during (C)SW-IMRT was high during the 6 weeks of treatment, but dropped afterwards. Exercise performance levels were low especially in patients who were treated with chemotherapy in combination with SW-IMRT.
